# State-of-the-art image and video quality assessment with a metric based on an intrinsically non-linear neural summation model

**DOI:** 10.3389/fnins.2023.1222815

**Published:** 2023-07-25

**Authors:** Raúl Luna, Itziar Zabaleta, Marcelo Bertalmío

**Affiliations:** ^1^Institute of Optics, Spanish National Research Council (CSIC), Madrid, Spain; ^2^Department of Information and Communication Technologies, Universitat Pompeu Fabra, Barcelona, Spain

**Keywords:** visual perception, visual neuroscience, receptive field, INRF, computational modeling, image quality assessment, video quality assessment, high frame rate videos

## Abstract

The development of automatic methods for image and video quality assessment that correlate well with the perception of human observers is a very challenging open problem in vision science, with numerous practical applications in disciplines such as image processing and computer vision, as well as in the media industry. In the past two decades, the goal of image quality research has been to improve upon classical metrics by developing models that emulate some aspects of the visual system, and while the progress has been considerable, state-of-the-art quality assessment methods still share a number of shortcomings, like their performance dropping considerably when they are tested on a database that is quite different from the one used to train them, or their significant limitations in predicting observer scores for high framerate videos. In this work we propose a novel objective method for image and video quality assessment that is based on the recently introduced Intrinsically Non-linear Receptive Field (INRF) formulation, a neural summation model that has been shown to be better at predicting neural activity and visual perception phenomena than the classical linear receptive field. Here we start by optimizing, on a classic image quality database, the four parameters of a very simple INRF-based metric, and proceed to test this metric on three other databases, showing that its performance equals or surpasses that of the state-of-the-art methods, some of them having millions of parameters. Next, we extend to the temporal domain this INRF image quality metric, and test it on several popular video quality datasets; again, the results of our proposed INRF-based video quality metric are shown to be very competitive.

## 1. Introduction

Image quality evaluation is of crucial importance in the media industry, where it has numerous practical applications, but also in applied disciplines such as image processing and computer vision, where it plays an important role in the development, optimization, and testing of algorithms. It may also be used to optimize any trade-offs between the components of an image/video transport system (i.e., video compression ratios, reserved network bandwidth) and perceived quality, aiming for a good user experience while maximizing the efficiency of the transmission, and thus reducing the ecological footprint associated to streaming. Subjective evaluation, consisting in measuring image quality by human beings, is costly and time-consuming. Therefore, the goal of objective quality assessment is to develop automatic methods that produce quantitative measures that are consistent with the perception of human observers. But this is a very challenging open problem in vision science, given the limitations of current visual perception models and the way they are exacerbated by emerging image display technologies of ever-increasing resolution, contrast, color gamut and framerate (Bertalmío, 2019).

Image quality methods can be divided into three categories: full-reference methods, which compare an original image with a distorted version of it; reduced-reference methods, that compare some characteristics of the distorted and reference image since the complete reference image is not available; and no-reference methods (also called blind models), operating solely on the distorted image.

In this article we will focus on full-reference methods, which constitute the vast majority of image quality approaches. A simple solution, and possibly the most widely used metric to estimate image quality, is the peak signal-to-noise ratio (PSNR), which is a non-linear transform of the mean square error (MSE) between the reference and the distorted images, another very popular metric. These metrics are simple to calculate, and they have a clear physical meaning; however, they are not very well correlated with perceived visual quality (Wang et al., 2004).

Therefore, in the last two decades, the goal of image quality assessment (IQA) research has been to improve these metrics by developing more sophisticated methods that mimic some aspects of the visual system. For instance, the Normalized Laplacian Pyramid Distance (NLPD) (Laparra et al., 2016) is based on transformations present in the early visual system: local luminance subtraction and local gain control, obtained from a decomposition of images using a Laplacian pyramid; the Structural Similarity Index (SSIM) (Wang et al., 2004) is based on the hypothesis that the human visual system is highly adapted for extracting structural information from the viewing field; the Feature Similarity Index (FSIM) (Zhang et al., 2011) is based on the assumption that the human visual system understands an image according to its low-level features, such as the phase congruency, which measures the significance of a local structure, and the image gradient magnitude, which encodes contrast information; the Visual Signal-to-Noise Ratio (VSNR) (Chandler and Hemami, 2007) analyses visual perception distortions in the wavelet domain; the Noise Quality Measure (NQM) (Damera-Venkata et al., 2000) is based on the contrast pyramid by Peli (1990); and the Visual Information Fidelity Measure (VIF) (Sheikh and Bovik, 2006) is based on natural scene statistics and models of the image degradation process and the human visual system. There are also learning-based methods that learn a metric from a set of training images and their corresponding perceptual scores. For instance, the Learned Perceptual Image Patch Similarity (LPIPS) metric (Zhang et al., 2018) is based on the hypothesis that perceptual similarity is a consequence of visual representations, as the authors found that internal activations of networks trained on high-level image classification tasks correspond well to human perceptual judgments; another example is the Deep Image Structure and Texture Similarity (DISTS) Metric (Ding et al., 2020), which uses a variant of the VGG convolutional neural network to construct a function that combines structure and texture similarity measurements between corresponding feature maps of the reference and distorted images; and PerceptNet (Hepburn et al., 2020), which is a convolutional neural network where the architecture reflects the structure and various stages in the human visual system: a cascade of canonical linear filters and divisive normalization layers simulate the retina-LGN-cortex pathway.

For video quality assessment (VQA), a simple option is to apply image quality metrics on a frame-by-frame basis, but this type of approach often provides a limited performance, especially in the case of high framerate (HFR) videos (Madhusudana et al., 2021). Therefore, the state-of-the-art in VQA are algorithms specifically developed for video, including the Spatio-temporal Reduced Reference Entropic Differences (ST-RRED) (Soundararajan and Bovik, 2012) or the Spatial Efficient Entropic Differencing for Quality Assessment (SpEED) (Bampis et al., 2017), both measuring quality deviations by computing spatial and temporal entropic differences in the band-pass domain; Frame Rate dependent Quality Metric (FRQM) (Zhang et al., 2017), which outputs quality measurements by calculating absolute differences between sequences that have been temporally filtered by a wavelet; Video Multi-method Assessment Fusion (VMAF) (Li et al., 2016), which, using a Support Vector Regressor, fuses a frame-difference feature with a detail feature and with features obtained from a Visual Information Fidelity (VIF) measure (Sheikh and Bovik, 2006); Deep Video Quality Assessor (deepVQA) (Kim et al., 2018), which combines a CNN model with a Convolutional Neural Aggregation Network (CNAN) used for temporal pooling; the Visual Quality Metric (VQM) (Pinson and Wolf, 2004), which uses reduced-reference technology (ITU-T, 2005) to provide estimates of video quality; the perceptual spatio-temporal frequency-domain based MOtion-based Video Integrity Evaluation (MOVIE) index (Seshadrinathan and Bovik, 2009), which monitors distortions that are perceptually relevant along motion trajectories; or the more recent Generalized Spatio-Temporal Index (GSTI) (Madhusudana et al., 2020), which calculates entropic differences between responses that have been temporally band-pass filtered.

Despite the notable advances in the field, it is important to point out that state-of-the-art quality assessment methods still share a number of shortcomings, like their performance dropping considerably when they are tested on a database that is quite different from the one used to train them (Ding et al., 2021), or their significant difficulties in predicting observer scores for HFR videos (Madhusudana et al., 2021). In this study we aim to overcome these limitations by estimating perceived image and video quality using a model for neural summation introduced recently, called the Intrinsically Non-linear Receptive Field (INRF) (Bertalmío et al., 2020) formulation; the INRF model successfully explains experimental data that linear receptive field models are unable to explain or do not explain accurately (Bertalmío et al., 2020), and it has been shown to be very promising as a tool to develop IQA methods given its ability to model complicated perceptual phenomena.

The main contributions of this work are as follows. Firstly, we start by optimizing, on a classic image quality database, the four parameters of a very simple INRF-based metric, and proceed to test this metric on three other databases, showing that its performance equals or surpasses that of the state-of-the-art IQA methods, some of them having millions of parameters. Secondly, we extend to the temporal domain this INRF image quality metric, and test it on several popular video quality databases; our results show that the proposed INRF-based VQA is very competitive, ranking best in several challenging scenarios like those provided by a very recent dataset for high frame rate videos. Finally, and to the best of our knowledge, the approach of using a neural summation model to create IQA and VQA methods is completely novel, and given its success it might pave the way for other neuroscience models to inform the design of new image quality assessment algorithms.

The structure of this manuscript is as follows. Section “2. Proposed methods for IQA and VQA” explains the INRF model and how it is used for IQA and VQA. Section “3. Materials and methods” provides details on how the INRF parameters are optimized for IQA and VQA usage, describes the different datasets on which performance is tested and explains further points on how the assessment of INRF for IQA and VQA models is performed. Section “4. Results and comparisons” shows the performance of INRF, employed for IQA and VQA, on different datasets, and results are compared with performance of other state-of-the-art IQA and VQA algorithms on those same datasets. Finally, in Section “5. Discussion,” results and their implications are discussed.

## 2. Proposed methods for IQA and VQA

### 2.1. Overview of the INRF neural summation model

In vision science, the receptive field (RF) of a neuron is the extent of the visual field where light influences the neuron’s response. In the “standard model” of vision, the first stage is a filtering operation consisting of multiplying the intensities at each local region of an image stimulus by the values of a filter (the weights of the RF), and summing the resulting intensities (Carandini et al., 2005); this weighted sum may then be normalized by the responses of neighboring neurons and passed through a point-wise non-linearity. Many scientists have come to accept this linear-plus-non-linear (L + NL) formulation as a working model of the visual system (Olshausen and Field, 2005), both in visual neuroscience and in visual perception, and while there have been considerable improvements on, and extensions to, the standard model, the linear RF remains as the foundation of most vision models. But there are a number of problems that are inherent to considering the RF as having a linear form, of which we will highlight three:

• Adaptation makes the linear RF change with the input and, in fact, the linear RF has been observed to have different sizes, orientations, preferred directions, or even different polarity (ON/OFF) for different stimuli (Cavanaugh et al., 2002; Coen-Cagli et al., 2012; Jansen et al., 2018);
• a linear RF depends on the choice of basis functions used to estimate it (Vilankar and Field, 2017);
• the linear RF is not supported by more recent neuroscience, and a growing number of neuroscience studies show that in general individual neurons *cannot* be modeled as a linear RF followed by an output non-linearity (Poirazi et al., 2003; Polsky et al., 2004; London and Häusser, 2005; Silver, 2010; Rodrigues et al., 2021).

In contrast, the INRF formulation is a physiologically plausible single-neuron summation model which, unlike the linear RF:

• Embodies the efficient representation principle and can remain constant in situations where the linear RF must change with the input;
• is a generalization of the linear RF that is much more powerful in representing non-linear functions;
• is consistent with more recent studies on dendritic computations.

The INRF equation for the response of a single neuron at location *x* (a set of 2D spatial coordinates, horizontal and vertical) is:


(1) $$I\,N\,R\,F\,\left(x\right)=\sum_{i}m_{i}\,I\,\left(y_{i}\right)-\lambda \,\sum_{i}w_{i}\,\sigma \,\left(I\,\left(y_{i}\right)-\sum_{j}g\,\left(y_{j}-x\right)\,I\,\left(y_{j}\right)\right)$$

where *m*_*i*_ stands for a 2D kernel *m*(*x*,*y*_*i*_), locations *y*_*i*_ (vertical and horizontal 2D spatial coordinates) are neighbors of *x*, λ is a scalar, *w*_*i*_ stands for a 2D kernel *w*(*x*,*y*_*i*_), σ represents a non-linearity, and *g* is also a 2D kernel.

The model is based on knowledge about dendritic activity: some dendritic branches act as non-linear units (Silver, 2010), a single non-linearity is not enough to model dendritic computations (Poirazi et al., 2003), and there is feedback from the neuron soma to the dendrites (London and Häusser, 2005). In the INRF model, some dendrites are linear and their contributions are summed with weights *m*_*i*_, and some other dendrites are non-linear and their contributions are summed with weights *w*_*i*_. The feedback from the soma is reflected in the shifting nature of the non-linearity σ, expressed by the term $\sum_{j}g\,\left(y_{j}-x\right)\,I\,\left(y_{j}\right)$, which has the effect of making the non-linearity change for each contributing location *y*_*i*_.

Using a single, fixed INRF module where the kernels *m*,*w*,*g* have Gaussian form and the non-linearity is a power-law sigmoid, and applying it to grayscale images, where *x* and *y*_*i*_ now denote pixel locations and *I*(⋅) are pixel values, the model response *INRF*(⋅) emulates the perceived image and can explain several visual perception phenomena that challenge the standard model (Bertalmío et al., 2020):

• The “crispening” effect. Brightness perception curves show “crispening” (slope increase) at the surround luminance level when the background is uniform, but the phenomenon disappears for salt and pepper background (Kane and Bertalmío, 2019). The INRF response qualitatively replicates both cases with a fixed set of parameters, which is not possible with a linear RF formulation.
• White’s illusion under noise. The INRF output qualitatively predicts the observers’ response to White’s illusion when bandpass noise is added, while in Betz et al. (2015) none of the vision models that were tried, based on linear RFs, were able to replicate this behavior.
• Light/dark asymmetry (the “*irradiation illusion*”). This phenomenon can be reproduced with a fixed INRF formulation, while a L + NL model needs to change with the stimulus (Kremkow et al., 2014).

In short, we could say that the INRF formulation, a non-linear transform of light intensity values, is a good estimator of brightness perception. This is a very valuable property when creating an image quality assessment method, as we discuss next.

### 2.2. IQA with the INRF model

Given that applying the INRF model to a grayscale image produces a result that appears to closely resemble how the image is perceived, Bertalmío et al. (2020) proposed a very simple image quality metric: given an image *I*, and its distorted version *I*_*D*_, the INRF transformation is applied to both of the images, obtaining *O* and *O*_*D*_, and then the root mean square error (RMSE) between the processed images is computed. The underlying idea here was that the INRF model performs a sort of “perceptual linearization,” a non-linear transform that brings *light-intensity* images (whose direct comparison, with metrics like RMSE or PSNR, does not correlate well with perception), to the space corresponding to *perceptual* images (which can now be compared with a simple Euclidean metric like RMSE). This metric had only five parameters that were optimized for the “crispening” brightness perception experiment mentioned above (with σ a power-law sigmoid and *g* a delta function) on a handful of synthetic images, and despite this fact and the simplicity of the metric, when tested on the natural image database TID2013 (Ponomarenko et al., 2015) it was shown to have a performance very similar to that of the state-of-the-art deep learning perceptual metric LPIPS (Zhang et al., 2018), with over 24 million parameters and close to 500K human judgments for labeling pair-comparison preferences on the 160,000 + natural images it used for training.

**FIGURE 1:**
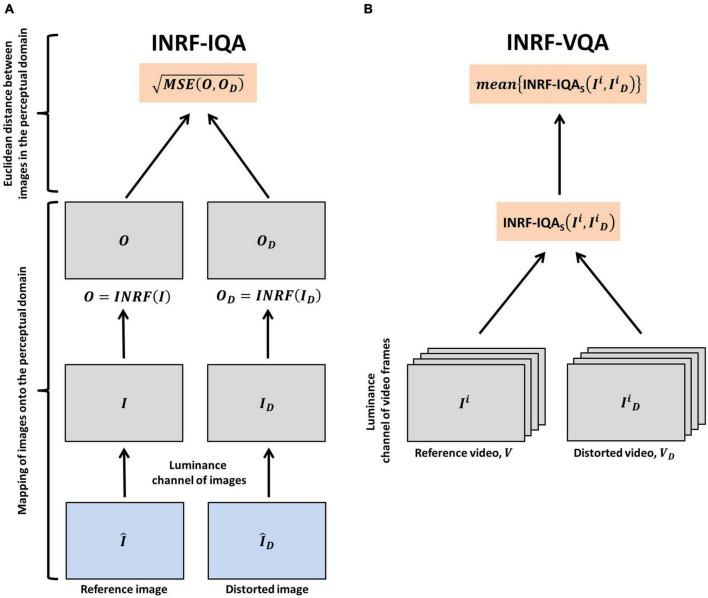
Schematic representation of how INRF-IQA and INRF-VQA are calculated. **(A)** Calculation of INRF-IQA. The luminance component of a reference image, $\hat{I}$, and a distorted image, ${\hat{I}}_{D}$, are mapped onto the perceptual domain by applying an INRF transformation on them: *O* and *O*_*D*_ are, respectively, obtained. Next, the Euclidean distance of the images in the perceptual domain is calculated by computing the root-mean-squared error of the INRF-transformed images. **(B)** Calculation of INRF-VQA. The process outlined in panel **(A)** is applied on a frame-wise basis to the luminance component of the video frames in a reference video, *V*, and a distorted video, *V*_*D*_. These video frames are referred to as *I^i^* and $I_{D}^{i}$, respectively. INRF-IQA is calculated using the reference video frames, *I^i^*, and the distorted video frames, $I_{D}^{i}$, that compose the reference and distorted videos, *V* and *V*_*D*_. The final INRF-VQA metric is obtained by computing the mean of the frame-wise INRF-IQA scores.

Based on this very promising result, here we propose a full-reference INRF-based IQA method in the following way (see Figure 1A for a graphical explanation of the process):

(1) Given a grayscale image *I*, the INRF transformation applied to it produces an image *O* whose value at each pixel location *x* is computed as:


(2) $$O\,\left(x\right)=I\,N\,R\,F\,\left(x\right)=m\,\text{⋆}\,I\,\left(x\right)-\lambda \,\sum_{i}w_{i}\,S\,\left(I\,\left(y_{i}\right)-g\,\text{⋆}\,I\,\left(x\right)\right)$$

where the kernels *m*,*w*,*g* are 2D Gaussians of standard deviations σ_*m*_,σ_*w*_,σ_*g*_, respectively, λ is a scalar, *S* is a sigmoid that has the form of an **atan** function, and the symbol ⋆ denotes the 2D convolution.

(2) The INRF-IQA value comparing image *I* and its distorted version *I*_*D*_ is computed as:


(3) $$I\,N\,R\,F-I\,Q\,A\,\left(\hat{I},{\hat{I}}_{D}\right)=\sqrt{M\,S\,E\,\left(O,O_{D}\right)}$$

$$=\sqrt{M\,S\,E\,\left(I\,N\,R\,F\,\left(I\right),I\,N\,R\,F\,\left(I_{D}\right)\right)}$$

where grayscale image *I* is the luminance channel of $\hat{I}$, grayscale image *I*_*D*_ is the luminance channel of ${\hat{I}}_{D}$, and the INRF transform of an image is computed as in Eq. 2.

(3) The values for the four parameters of the metric, namely σ_*m*_,σ_*w*_,σ_*g*_, and λ, are chosen so as to maximize the correlation between the INRF-IQA perceptual distance of Eq. 3 and the mean opinion scores (MOS) of human observers over the large-scale natural image database TID2008 (Ponomarenko et al., 2009) (see section “3. Materials and methods”).

We must note that the IQA method in Bertalmío et al. (2020) used a different non-linearity (a power-law sigmoid instead of the atan function), considered *g* to be a delta function instead of a 2D kernel, and used as parameter values the same set that allowed the INRF model to reproduce the results of certain brightness perception experiment.

### 2.3. VQA with the INRF model

We propose as a full-reference INRF-based VQA metric a straightforward combination of the INRF-IQA metric of Eq. 3 with a simple temporal pooling strategy: the image metric is applied frame-by-frame, and then the results are averaged (see Figure 1B for a graphical explanation of the process). That is, given a reference video *V* and a distorted version *V*_*D*_, the output of our proposed INRF-VQA metric is:


(4) $$I\,N\,R\,F-V\,Q\,A\,\left(V,V_{D}\right)=m\,e\,a\,n\,\left\{I\,N\,R\,F-I\,Q\,A_{s}\,\left(I^{i},I_{D}^{i}\right)\right\}$$

where *I^i^* and $I_{D}^{i}$ are, respectively, the luminance channel of the i-th frame of *V* and *V*_*D*_, and the IQA metric *INRF*−*IQA*_*s*_ has the same form as in Eq. 3 and the same value of *3* for the parameter λ, but the spatial kernel sizes σ_*m*_,σ_*w*_,σ_*g*_ are scaled by a factor *f* that denotes the ratio between the size of the frames in the video *V* and the size of the images in TID2008 (that were used to optimize σ_*m*_,σ_*w*_,σ_*g*_). For instance, if *V* is a 2K video of resolution 1920×1080, and given that images in TID2008 are of size 512×384, the scaling factor is $f=\frac{1920}{512}=3.75$.

## 3. Materials and methods

### 3.1. Optimization details

The parameter values of INRF-IQA (Eq. 3) are found through optimization on the TID2008 dataset (Ponomarenko et al., 2009) to maximize the Pearson correlation coefficient (PLCC) between the observers’ scores for the images in TID2008 and the corresponding INRF-IQA scores. In particular, a grid search optimization method is used to optimize the INRF-IQA parameter values σ_*m*_,σ_*w*_,σ_*g*_, and λ, and the optimal parameters found are: σ_*m*_ = 1.74,σ_*w*_ = 25,σ_*g*_ = 1 and λ = 3.

### 3.2. IQA datasets

We use the LIVE (Sheikh et al., 2006), CSIQ and TID2013 (Ponomarenko et al., 2015) datasets to test the performance of our INRF-IQA metric on images; each dataset also containing subjective image quality scores from observers for those same images. The LIVE dataset consists of 779 images. It has a total of 29 reference images whose distorted versions are achieved by applying 5 different types of distortion (Gaussian blur, additive white Gaussian noise, JPEG compression, JP2K compression and a simulated fast fading Rayleigh channel) with different distortion levels. The CSIQ dataset has 866 images, with 30 being reference ones. Their distorted versions are obtained through Gaussian blur, Gaussian pink noise, Gaussian white noise, JPEG compression, JP2K compression and contrast change. The TID2013 dataset extends the previous TID2008 one (Ponomarenko et al., 2009). It is composed of 3,000 images, out of which 25 are reference ones. These are distorted to achieve the rest of the images by applying 24 distortion types (7 new types of distortions with respect to TID2008) each with 5 distortion levels. Distortion types are rich, spanning from Gaussian noise, Gaussian blur, lossy compression of noisy images, and distortions such as JPEG to more uncommon distortions like non-eccentricity pattern noise.

### 3.3. VQA datasets

We test the performance of our INRF-VQA model on four popular video quality databases, all publicly available and containing observers’ scores for a number of common spatial and temporal distortions: LIVE-YT-HFR, LIVE-MOBILE, LIVE-VQA and VQEG-HD3.

The very recent LIVE-YT-HFR dataset (Madhusudana et al., 2021) spans 16 different video categories, each showing a different progressively scanned natural scene. Out of those 16 contents, 11 have 2K spatial resolution and 5 have 4K resolution. Within each of the video contents, videos with different frame rates exist, out of which we use those of 120, 60, and 30 fps. Each video content with a given frame rate has 5 possible compression levels [FFmpeg VP9 compression (Mukherjee et al., 2015), and single-pass encoding varying the Constant Rate Factor (CRF)]. For instance, a given content and its videos with a given frame rate sum a total of 5 videos: 1 video with lossless compression (CRF = 0) plus 4 videos with compression levels ranging from CRF = 4 to CRF = 63. Finally, for each video content, the 120 fps video with lossless compression (CRF = 0) is referred to as the reference sequence. All the remaining videos within a content (that is, 120 fps videos with CRF values larger than 0, and 60 fps and 30 fps videos), are the distorted sequences.

The LIVE-MOBILE dataset (Moorthy et al., 2012a,b,c) consists of 12 reference videos with frame rates of 30 fps and a spatial resolution of 1280 × 720 pixels over which different distortion types are applied to produce distorted videos. The existing distortion types are: H 0.264 compression at four different rates; wireless channel packet-loss distortion; freeze-frames where a loss of temporal continuity exists after freeze and where no such loss of temporal continuity takes place (not used in the analyses); rate adaptation distortion (i.e., compression rate dynamically varies between two compression rates); and temporal dynamics (compression rate is varied between several rates with different rate-switching structures). Subjective measurements acquired through viewing the different videos on a small mobile screen are available.

The LIVE-VQA dataset (Seshadrinathan et al., 2010a,b) consists of 10 reference videos with a frame rate of 25 or 50 fps and a spatial resolution of 768 × 432 pixels. The existing distortion types are MPEG2 compression, H 0.264 compression, simulated transmission through error-prone IP networks and simulated transmission through error-prone wireless networks.

Finally, the VQEG-HD3 dataset (Video Quality Experts Group, 2010) consists of 13 reference videos with a frame rate of 30 fps and a spatial resolution of 1920 × 1080 pixels. Distorted videos are achieved by applying the distortion levels hrc04, hrc07, hrc16, hrc17, hrc18, hrc19, hrc20, and hrc21.

### 3.4. Evaluation of INRF-IQA and INRF-VQA models

Existing subjective image quality and video quality scores in each of the datasets are, respectively, correlated with our INRF-IQA and INRF-VQA metrics. In the case of INRF-IQA, SRCC is calculated. For INRF-VQA, SRCC and PLCC are obtained. Before calculating PLCC, predicted objective video quality scores are passed through a four-parameter sigmoid function as described in Antkowiak et al. (2000):


(5) $$\hat{y}\,\left(x\right)=\beta_{2}\,\frac{\beta_{1}-\beta_{2}}{1+e^{-\left(\frac{x-\beta_{3}}{\left|\beta_{4}\right|}\right)}}$$

where *x* stands for the raw INRF-VQA scores, and β_1_,β_2_,β_3_, and β_*4*_ are its parameters $\hat{y}$.

## 4. Results and comparisons

### 4.1. IQA

Performance of our INRF-IQA metric is evaluated on images from the LIVE (Sheikh et al., 2006), CSIQ and TID2013 (Ponomarenko et al., 2015) datasets, and compared with that of other full-reference IQA methods; results are shown in Table 1. The performance of the methods PerceptNet and LPIPS is shown in three different training scenarios: (1) training performed on the ImageNet (Deng et al., 2009) and BAPSS (Zhang et al., 2018) datasets, (2) training performed only on the BAPPS dataset, and (3) training performed on the TID2008 dataset (Ponomarenko et al., 2009). None of the IQA methods was tested on a dataset on which it had been specifically trained. GMSD needs tuning of one parameter, with its value selected so as to provide maximum performance in the three datasets considered. We can see that the performance of INRF-IQA is consistently very good across all datasets, surpassing CNN-based models such as LPIPS (which has 24.7 million parameters) (Zhang et al., 2018) and DISTS (Ding et al., 2020), as well as other extensively used classical methods like NLPD (Laparra et al., 2016). Overall, the best IQA performance is observed for PerceptNet (Hepburn et al., 2020) when its 36.3 thousand parameters are optimized for TID2008 (not for BAPPS or ImageNet), GMSD (Xue et al., 2014) and our INRF-IQA metric (with 4 parameters).

**TABLE 1:** Numbers indicate spearman rank correlation coefficients (SRCC).

	LIVE	CSIQ	TID2013	(Mean)
MS-SSIM	0.951	0.886	0.782	(0.873)
CW-SSIM	0.781	0.738	0.680	(0.733)
VIF	**0.963**	0.911	0.676	(0.850)
NLPD	0.938	0.937	0.800	(0.892)
GMSD	0.960	**0.950**	**0.804**	**(0.905)**
MAD	0.960	0.941	0.773	(0.891)
FSIM	**0.963**	0.916	**0.802**	(0.894)
VSI	0.950	0.923	0.793	(0.889)
DISTS	0.942	0.905	0.764	(0.870)
LPIPS_A_	0.96	0.93	0.76	(0.883)
LPIPS_B_	0.89	0.80	0.57	(0.753)
PerceptNet_A_	0.94	0.88	0.76	(0.86)
PerceptNet_B_	0.93	0.84	0.72	(0.83)
PerceptNet_C_	**0.98**	**0.96**	**0.87**	**(0.936)**
INRF-IQA	0.947	**0.952**	**0.802**	**(0.900)**

The INRF-IQA metric is compared against a set of full-reference image quality methods: MS-SSIM (Wang et al., 2003), CW-SSIM (Wang and Simoncelli, 2005), VIF (Sheikh and Bovik, 2006), NLPD (Laparra et al., 2016), GMSD (Xue et al., 2014), MAD (Larson and Chandler, 2010), FSIM (Zhang et al., 2011), VSI (Zhang et al., 2014), DISTS (Ding et al., 2020), LPIPS (Zhang et al., 2018), and PerceptNet (Hepburn et al., 2020). LPIPS_A_ and PerceptNet_A_ are trained on the ImageNet (Deng et al., 2009) and BAPSS (Zhang et al., 2018) datasets, and LPIPS_B_ and PerceptNet_B_ are trained only on the BAPPS dataset. PerceptNetC is trained on the TID2008 dataset (Ponomarenko et al., 2009). The best three correlation values per column are marked in bold. Adapted table from Hepburn et al. (2020) and Ding et al. (2021).

### 4.2. VQA

We start evaluating the performance of our INRF-VQA metric on videos from the very recent (and challenging) LIVE-YT-HFR dataset (Madhusudana et al., 2021). This dataset consists of reference videos of 120 fps frame rate for which distorted versions are generated by reducing their frame rate and applying different compression levels. Subjective measurements of video quality are provided for each of the videos.

It is important to note that our INRF-VQA metric, as well as many other full-reference metrics, needs reference and distorted video sequences to have the same number of frames. For this reason, when a distorted video has a lower frame rate than the reference video, either the reference video must be downsampled to match the number of frames in the distorted video or the distorted video must be upsampled to match the reference. Madhusudana et al. (2021) (see their Table 5), who test the performance of several VQA algorithms on the LIVE-YT-HFR dataset, use naive temporal upsampling by frame duplication of distorted videos. In doing so, they argue that downsampling may introduce undesired temporal artifacts in reference videos. SSIM (Wang et al., 2004), FSIM (Zhang et al., 2011) and VMAF (Li et al., 2016) are very successful, state-of-the-art, full-reference VQA metrics, and their performance comparison against our INRF-VQA metric can be seen in Table 2. Performance is shown for different frame rates in the LIVE-YT-HFR dataset: 120, 60, and 30 fps; and in the case of 60 and 30 fps, both an upsampling (results taken from Madhusudana et al., 2021) and a downsampling approach are used. Upsampling is achieved by frame duplication of distorted videos and downsampling is done through frame dropping of reference videos. This way, reference and distorted videos have the same number of frames. Spearman and Pearson correlation coefficients (SRCC and PLCC) of the objective VQA metrics with subjective video quality scores are displayed.

**TABLE 2:** Numbers indicate SRCC and PLCC for frame rates of 120, 60, and 30 fps in the LIVE-YT-HFR dataset.

	120 fps		60 fps		30 fps	
	SRCC	PLCC	SRCC	PLCC	SRCC	PLCC
SSIM_dup_ SSIM_drop_	0.7485	0.6726	0.2123	0.1845	0.1108	0.0816
			0.5500	0.5077	0.5718	0.5844
FSIM_dup_ FSIM_drop_	0.7053	0.6368	0.3450	0.2776	0.2487	0.1786
			0.6197	0.6585	0.5984	0.7524
VMAF_dup_ VMAF_drop_	0.7943	0.7844	0.5408	0.6015	0.2855	0.3740
			0.6664	0.8122	0.5981	0.8713
INRF-VQA_dup_ INRF-VQA_drop_	0.8507	0.8261	0.2983	0.2905	0.1198	0.2324
			0.7525	0.8262	0.6331	0.8750

The INRF-VQA metric is compared against SSIM (Wang et al., 2004), FSIM (Zhang et al., 2011), and VMAF (Li et al., 2016). For 60 and 30 fps, SSIM, FSIM, VMAF, and INRF-VQA results are shown both using frame duplication of distorted videos (SSIM_dup_, FSIM_dup_, VMAF_dup_, and INRF-VQA_dup_, respectively) and frame dropping of reference videos (SSIM_drop_, FSIM_drop_, VMAF_drop_, and INRF-VQA_drop_). SSIM_dup_, FSIM_dup_, and VMAF_dup_ results are taken from Madhusudana et al. (2021), their Table 5.

For 60 and 30 fps, and for the four VQA methods, performance is seen to improve when a frame dropping strategy rather than a duplication one is used (for which correlations are low). For this reason, we use this approach as our preferred choice to evaluate INRF-VQA for 30 and 60 fps.

Table 3 shows a performance comparison of our INRF-VQA metric (using frame dropping when 60 and 30 fps videos are evaluated) against other state-of-the-art full-reference VQA metrics. SRCC and PLCC results of the objective VQA metrics with subjective video quality scores are shown for different frame rates in the LIVE-YT-HFR dataset.

**TABLE 3:** Numbers indicate SRCC and PLCC for frame rates of 120, 60, and 30 fps in the LIVE-YT-HFR dataset.

	120 fps		60 fps		30 fps	
	SRCC	PLCC	SRCC	PLCC	SRCC	PLCC
PSNR	0.6019	0.5937	**0.6202**	0.5719	0.4414	0.4179
SSIM	**0.7485**	0.6726	0.5500	0.5077	0.5718	0.5844
MS-SSIM	0.6165	0.5843	0.2516	0.1900	0.1929	0.1112
FSIM	0.7053	0.6368	0.6197	0.6585	**0.5984**	0.7524
ST-RRED	0.6745	0.5906	0.5062	0.4457	0.1188	0.0307
SpEED	0.6827	0.6097	0.1824	0.1110	0.2278	0.0896
FRQM	–	–	0.0947	0.0309	0.0983	0.0854
VMAF	**0.7943**	**0.7844**	**0.6664**	**0.8003**	**0.5981**	**0.8713**
deepVQA	0.6865	0.6209	0.2527	0.1652	0.1353	0.1059
GSTI	0.7390	**0.7003**	0.6015	**0.7566**	0.4758	**0.6689**
INRF-VQA	**0.8507**	**0.8261**	**0.7525**	**0.8262**	**0.6331**	**0.8750**

The INRF-VQA metric is compared against a set of full-reference video quality methods: PSNR, SSIM (Wang et al., 2004), MS-SSIM (Wang et al., 2003), FSIM (Zhang et al., 2011), ST-RRED (Soundararajan and Bovik, 2012), SpEED (Bampis et al., 2017), FRQM (Zhang et al., 2017), VMAF (Li et al., 2016), deepVQA (Kim et al., 2018), and GSTI (Madhusudana et al., 2020). For 60 and 30 fps, results for all VQA algorithms are shown using naive temporal upsampling of distorted videos except for INRF-VQA, SSIM, FSIM, and VMAF, where frame dropping of reference videos is used. The best three correlation values per column are marked in bold. Adapted from Madhusudana et al. (2021).

The results in Table 3 demonstrate that INRF-VQA consistently outperforms all state-of-the-art algorithms for all frame rates tested, including the highest frame rate of 120 fps (for a fair comparison, results for SSIM, FSIM and VMAF for 60 and 30 fps were also computed using frame dropping, and we do not rule out that other methods could also benefit from carrying out the validation in this manner).

Next, we evaluate our INRF-VQA metric on a very popular dataset, the LIVE-MOBILE (Moorthy et al., 2012a,b,c). Table 4 shows, for several popular metrics and for INRF-VQA, the results for the different distortion types as well as the overall performance. As we can see, INRF-VQA ranks among the best-performing metrics for most distortions.

**TABLE 4:** Numbers indicate SRCC and PLCC for the different distortion types in the LIVE-MOBILE dataset: compression, rate adaptation, temporal dynamics and wireless; as well as global performance.

	Compression		Rate adaptation		Temporal dynamics		Wireless		All	
	SRCC	PLCC	SRCC	PLCC	SRCC	PLCC	SRCC	PLCC	SRCC	PLCC
PSNR	0.8185	0.7841	0.5981	0.5364	0.3717	0.4166	0.7925	0.7617	0.6780	0.6909
SSIM	0.7092	0.7475	0.6303	0.6120	0.3429	0.3924	0.7246	0.7307	0.6498	0.6637
MS-SSIM	0.8044	0.7664	**0.7378**	**0.7089**	**0.3974**	0.4068	0.8128	0.7706	0.7425	0.7077
FSIM	**0.8896**	0.8368	0.6269	0.5223	0.3020	0.2674	**0.8849**	0.8421	0.7491	0.7230
SpEED	**0.9418**	**0.9379**	**0.7262**	**0.7807**	**0.4214**	**0.5697**	**0.9390**	**0.9391**	**0.8051**	**0.8118**
VSNR	0.8739	0.8489	0.6735	0.6581	0.3170	**0.4269**	0.8559	0.8493	**0.7517**	0.7592
VIF	0.8613	**0.8826**	0.6388	0.6643	0.1242	0.1046	0.8739	**0.8979**	0.7439	**0.7870**
UQI	0.5621	0.5794	0.4299	0.2929	0.0296	0.2546	0.5756	0.7412	0.4894	0.6619
NQM	0.8499	0.8318	0.6775	0.6772	0.2383	0.3646	**0.8985**	0.8738	0.7493	0.7622
WSNR	0.7817	0.7558	0.5598	0.5365	0.0942	0.0451	0.7510	0.7276	0.6267	0.6320
SNR	0.7073	0.6501	0.5565	0.3988	0.2029	0.0839	0.6959	0.6052	0.5836	0.5189
VQM	0.7717	0.7816	0.6475	0.5910	0.3860	0.4066	0.7758	0.7909	0.6945	0.7023
MOVIE	0.7738	0.8103	**0.7198**	**0.6811**	0.1578	0.2436	0.6580	0.7266	0.6420	0.7157
INRF-VQA	**0.8891**	**0.8868**	0.7137	0.6149	**0.5314**	**0.5834**	0.8840	**0.8916**	**0.7701**	**0.7684**

The INRF-VQA metric is compared against a set of full-reference video quality methods: PSNR, SSIM (Wang et al., 2004), MS-SSIM (Wang et al., 2003), FSIM (Zhang et al., 2011), SpEED (Bampis et al., 2017), VSNR (Chandler and Hemami, 2007), VIF (Sheikh and Bovik, 2006), UQI (Wang and Bovik, 2002), NQM (Damera-Venkata et al., 2000), Weighted Signal-to-Noise Ratio (WSNR), Signal-to-Noise Ratio (SNR), VQM (Pinson and Wolf, 2004), and MOVIE (Seshadrinathan and Bovik, 2009). The best three correlation values per column are marked in bold. Adapted from Moorthy et al. (2012a), except for FSIM and SpEED values.

**TABLE 5:** Numbers indicate SRCC and PLCC results in the LIVE-VQA (Seshadrinathan et al., 2010a,b) and VQEG-HD3 (Video Quality Experts Group, 2010) datasets.

	LIVE-VQA		VQEG-HD3	
	SRCC	PLCC	SRCC	PLCC
PSNR	0.5233	0.5489	0.7172	0.7212
SSIM	0.5251	0.4997	0.6841	0.6938
MS-SSIM	**0.7321**	**0.7387**	0.8567	**0.8640**
FSIM	**0.7317**	**0.7011**	**0.8915**	**0.8812**
SpEED	**0.7250**	**0.7147**	**0.8646**	0.8538
VSNR	0.6725	0.6883	0.7707	0.7763
VIF	0.5541	0.5682	0.6785	0.6920
UQI	0.4370	0.4493	0.6218	0.6283
NQM	0.6448	0.6687	0.6957	0.6932
WSNR	0.6410	0.6739	0.6862	0.6991
SNR	0.5580	0.6027	0.6294	0.6374
INRF-VQA	0.6697	0.6916	**0.8870**	**0.8914**

The INRF-VQA metric is compared against a set of full-reference video quality methods obtained from the Metrix Mux toolbox (Murthy and Karam, 2010): PSNR, SSIM (Wang et al., 2004), MS-SSIM (Wang et al., 2003), FSIM (Zhang et al., 2011), SpEED (Bampis et al., 2017), VSNR (Chandler and Hemami, 2007), VIF (Sheikh and Bovik, 2006), UQI (Wang and Bovik, 2002), NQM (Damera-Venkata et al., 2000), weighted signal-to-noise ratio (WSNR) and signal-to-noise ratio (SNR). The best three correlation values per column are marked in bold.

To further test our INRF-VQA metric, we evaluate its performance on two other popular video quality datasets widely used in the VQA literature, LIVE-VQA (Seshadrinathan et al., 2010a,b) and VQEG-HD3 (Video Quality Experts Group, 2010). Table 5 shows the correlation values in these datasets for INRF-VQA and for several popular metrics [PSNR, SSIM, MS-SSIM, VSNR, VIF, UQI, NQM, WSNR, and SNR are available in the Metrix Mux toolbox (Murthy and Karam, 2010)]. As we can see from these results, INRF-VQA performs very well in VQEGHD3 and shows a competitive performance in LIVE-VQA.

As a final note, we would like to remark that our INRF-VQA metric had its parameters optimized on an image dataset rather than a video dataset: this is also the approach followed by several of the best-performing VQA methods, like MS-SSIM, VIF, VSNR or NQM, while other excellent VQA algorithms are specifically trained on video quality datasets, like VMAF or GSTI.

## 5. Discussion

In this study we have taken a recent neural summation model and used it as a foundation for novel metrics for image and video quality assessment. To the best of our knowledge this is a novel approach, that might pave the way for other neuroscience models to inform the creation of IQA and VQA methods.

Our validation, on popular datasets of observer scores, shows that our proposed metrics for IQA and VQA compare very well with the state-of-the-art and, very importantly, that their performance is very good and does not drop substantially for different datasets, unlike what many methods are prone to do and is often the case with those based on deep learning techniques.

For the very recent, and challenging, video quality dataset LIVE-YT-HFR (Madhusudana et al., 2021), our metric for VQA is shown to outperform all state-of-the-art models, often by a wide margin, for all frame rates considered, including a high frame rate of 120 fps. Arguably, the distortions caused by the changes in frame rate in the LIVE-YT-HFR dataset are much less perceptually relevant than the artifacts created by compression: this would explain why the full-reference metrics not specifically designed to work with different frame rates (such as SSIM, FSIM, and INRF-VQA) correlate well with the observers’ responses even when the information about the temporal differences is reduced (i.e., when reference frames are dropped); on the other hand, frame dropping may prevent the metrics to fully capture the potential impact of different frame rates on the perceived quality. However, we also believe that the use of frame duplication may have implications for the performance evaluation of different methods. When distorted videos have a lower framerate than reference ones, and their number of frames is matched through temporal upsampling by frame duplication, duplicated frames from the distorted video are compared to frames from the reference video that are spatially shifted one from another (whenever there is motion in a video). Methods like PSNR, with a limited performance, may indicate perceptual differences in these cases that may not be especially sensitive to the aforementioned spatial shift. However, better performing algorithms like FSIM, SSIM, VMAF, or INRF-VQA itself, are sensitive toward this spatial shift. This translates into perceptual frame-difference judgments that, although accurate, are solely the result of the frame duplication strategy, and do not reflect human perception of quality. In short, the lower sensitivity of PSNR to the spatial shifts introduced by upsampling may paradoxically translate into a better performance than that of state-of-the-art algorithms which are sensitive to the artificially introduced spatial shifts.

It is important to remark that the parameters of our proposed INRF-VQA metric were optimized for image data, i.e., INRF-VQA was not trained on any video dataset. INRF-VQA is a straightforward extension of the INRF-IQA method, in which metric values are computed on a frame-by-frame basis and then averaged over time to produce a single score for the video: the fact that this simple temporal extension of an IQA method works so remarkably well for VQA challenges a common assumption in the literature, where it is thought that the best VQA metrics must be developed specifically for video Madhusudana et al. (2021).

Regarding future work, we want to point out that for INRF-VQA we have resorted to very simple and very effective design choices, like mean average for temporal pooling instead of a more optimal strategy (e.g., Rimac-Drlje et al., 2009), or the fact that computations on a present frame are not influenced by past frames. For this reason, we believe that embedding temporal processing into our INRF-VQA model in a way that is more biologically realistic could prove better. As well, we would like to deepen into the study of how stacking several INRF modules produces an increase in quality prediction performance. Positive IQA results in this regard have already been reported in Bertalmío et al. (2020). We are also interested in extending both INRF-IQA and INRF-VQA so that they consider color and can be applied to high dynamic range (HDR), wide color gamut (WCG) and 8K imagery.

## Data availability statement

The raw data supporting the conclusions of this article will be made available by the authors, without undue reservation. MATLAB code with a numerical implementation of the INRF-IQA/INRF-VQA algorithm is available at https://github.com/raullunadelvalle/INRF-IQA-VQA.git.

## Author contributions

RL and MB wrote the manuscript. RL did the final version of the INRF-VQA code and carried out the INRF-VQA experiments. IZ did the INRF-IQA version of the code and carried out the INRF-IQA experiments. MB conceptualized the original idea. All authors reviewed the manuscript.

## References

[B1] Antkowiak J. Baina T. J. Baroncini F. V. Chateau N. FranceTelecom F. Pessoa A. C. F. (2000). *Final report from the video quality experts group on the validation of objective models of video quality assessment.* Geneva: ITU-T Standards Contributions COM.

[B2] Bampis C. G. Gupta P. Soundararajan R. Bovik A. C. (2017). Speed-QA: Spatial efficient entropic differencing for image and video quality. *IEEE Signal Process. Lett.* 24 1333–1337. 10.1109/LSP.2017.2726542

[B3] Bertalmío M. (2019). *Vision models for high dynamic range and wide colour gamut imaging: Techniques and applications.* Cambridge, MA: Academic Press. 10.1016/B978-0-12-813894-6.00015-6

[B4] Bertalmío M. Gomez-Villa A. Martın A. Vazquez-Corral J. Kane D. Malo J. (2020). Evidence for the intrinsically nonlinear nature of receptive fields in vision. *Sci. Rep.* 10:16277. 10.1038/s41598-020-73113-0 33004868 PMC7530701

[B5] Betz T. Shapley R. Wichmann F. A. Maertens M. (2015). Testing the role of luminance edges in White’s illusion with contour adaptation. *J. Vis.* 15:14. 10.1167/15.11.14 26305862 PMC6897287

[B6] Carandini M. Demb J. B. Mante V. Tolhurst D. J. Dan Y. Olshausen B. A. (2005). Do we know what the early visual system does? *J. Neurosci.* 25 10577–10597. 10.1523/JNEUROSCI.3726-05.2005 16291931 PMC6725861

[B7] Cavanaugh J. R. Bair W. Movshon J. A. (2002). Selectivity and spatial distribution of signals from the receptive field surround in macaque V1 neurons. *J. Neurophysiol.* 88 2547–2556. 10.1152/jn.00693.2001 12424293

[B8] Chandler D. M. Hemami S. S. (2007). VSNR: A wavelet-based visual signal-to-noise ratio for natural images. *IEEE Trans. Image Process.* 16 2284–2298. 10.1109/tip.2007.901820 17784602

[B9] Coen-Cagli R. Dayan P. Schwartz O. (2012). Cortical surround interactions and perceptual salience via natural scene statistics. *PLoS Comput. Biol.* 8:e1002405. 10.1371/journal.pcbi.1002405 22396635 PMC3291533

[B10] Damera-Venkata N. Kite T. D. Geisler W. S. Evans B. L. Bovik A. C. (2000). Image quality assessment based on a degradation model. *IEEE Trans. Image Process.* 9 636–650. 10.1109/83.841940 18255436

[B11] Deng J. Dong W. Socher R. Li L. J. Li K. Fei-Fei L. (2009). “Imagenet: A large-scale hierarchical image database,” in *In 2009 IEEE conference on computer vision and pattern recognition*, (Miami, FL: IEEE), 248–255. 10.1109/CVPR.2009.5206848

[B12] Ding K. Ma K. Wang S. Simoncelli E. P. (2020). Image quality assessment: Unifying structure and texture similarity. *IEEE Trans. Pattern Anal. Mach. Intell.* 44 2567–2581. 10.1109/TPAMI.2020.3045810 33338012

[B13] Ding K. Ma K. Wang S. Simoncelli E. P. (2021). Comparison of full-reference image quality models for optimization of image processing systems. *Int. J. Comput. Vis.* 244 1258–1281. 10.1007/s11263-020-01419-7 33495671 PMC7817470

[B14] Hepburn A. Laparra V. Malo J. McConville R. Santos-Rodriguez R. (2020). “Perceptnet: A human visual system inspired neural network for estimating perceptual distance,” in *2020 IEEE International Conference on Image Processing (ICIP)* (IEEE), (Abu Dhabi: IEEE), 121–125. 10.1111/ejn.13725

[B15] ITU-T (2005). *User Requirements for Objective Perceptual Video Quality Measurements in Digital Cable Television.* Geneva: ITU-T Recommendation.

[B16] Jansen M. Jin J. Li X. Lashgari R. Kremkow J. Bereshpolova Y. (2018). Cortical balance between on and off visual responses is modulated by the spatial properties of the visual stimulus. *Cereb. Cortex* 29 336–355. 10.1093/cercor/bhy221 30321290 PMC6294412

[B17] Kane D. Bertalmío M. (2019). A reevaluation of Whittle (1986, 1992) reveals the link between detection thresholds, discrimination thresholds, and brightness perception. *J. Vis.* 19 16–16. 10.1167/19.1.16 30694300

[B18] Kim W. Kim J. Ahn S. Kim J. Lee S. (2018). “Deep video quality assessor: From spatio-temporal visual sensitivity to a convolutional neural aggregation network,” in *Proceedings of the European Conference on Computer Vision (ECCV)*, (Munich: Springer Verlag), 219–234. 10.1007/978-3-030-01246-5_14

[B19] Kremkow J. Jin J. Komban S. J. Wang Y. Lashgari R. Li X. (2014). Neuronal nonlinearity explains greater visual spatial resolution for darks than lights. *Proc. Natl. Acad. Sci. U. S. A.* 111 3170–3175. 10.1073/pnas.1310442111 24516130 PMC3939872

[B20] Laparra V. Ballé J. Berardino A. Simoncelii E. P. (2016). “Perceptual image quality assessment using a normalized Laplacian pyramid,” in *Human Vision and Electronic Imaging 2016, HVEI 2016*, (Virginia: Society for Imaging Science and Technology), 43–48.

[B21] Larson E. C. Chandler D. M. (2010). Most apparent distortion: Full-reference image quality assessment and the role of strategy. *J. Elect. Imaging* 19:011006. 10.1117/1.3267105

[B22] Li Z. Aaron A. Katsavounidis I. Moorthy A. Manohara M. (2016). Toward a practical perceptual video quality metric. *Netflix Tech. Blog*. 6:2.

[B23] London M. Häusser M. (2005). Dendritic computation. *Annu. Rev. Neurosci.* 28 503–532. 10.1146/annurev.neuro.28.061604.135703 16033324

[B24] Madhusudana P. C. Birkbeck N. Wang Y. Adsumilli B. Bovik A. C. (2020). Capturing video frame rate variations via entropic differencing. *IEEE Signal Process. Lett.* 27 1809–1813. 10.1109/LSP.2020.3028687

[B25] Madhusudana P. C. Yu X. Birkbeck N. Wang Y. Adsumilli B. Bovik A. C. (2021). Subjective and objective quality assessment of high frame rate videos. *IEEE Access* 9 108069–108082. 10.1109/ACCESS.2021.3100462 36772550

[B26] Moorthy A. K. Choi L. K. Bovik A. C. De Veciana G. (2012a). Video quality assessment on mobile devices: Subjective, behavioral and objective studies. *IEEE J. Select. Top. Signal Process.* 6 652–671. 10.1109/JSTSP.2012.2212417

[B27] Moorthy A. K. Choi L. K. De Veciana G. Bovik A. (2012b). Mobile video quality assessment database. *IEEE ICC Workshop Real. Adv. Video Opt. Wireless Netw.* 6 652–671.

[B28] Moorthy A. K. Choi L. K. De Veciana G. Bovik A. C. (2012c). “Subjective analysis of video quality on mobile devices,” in *Sixth International Workshop on Video Processing and Quality Metrics for Consumer Electronics (VPQM)*, (Scottsdale).

[B29] Mukherjee D. Han J. Bankoski J. Bultje R. Grange A. Koleszar J. (2015). A technical overview of vp9—the latest open-source video codec. *SMPTE Motion Imaging J.* 124 44–54. 10.5594/j18499

[B30] Murthy A. V. Karam L. J. (2010). “A MATLAB-based framework for image and video quality evaluation,” in *2010 Second International Workshop on Quality of Multimedia Experience (QoMEX)*, (Piscataway, NJ: IEEE), 242–247. 10.1109/QOMEX.2010.5516091

[B31] Olshausen B. A. Field D. J. (2005). How close are we to understanding V1? *Neural Comput.* 17 1665–1699. 10.1162/0899766054026639 15969914

[B32] Peli E. (1990). Contrast in complex images. *JOSA A* 7 2032–2040. 10.1364/JOSAA.7.002032 2231113

[B33] Pinson M. H. Wolf S. (2004). A new standardized method for objectively measuring video quality. *IEEE Trans. Broadcast.* 50 312–322.

[B34] Poirazi P. Brannon T. Mel B. W. (2003). Pyramidal neuron as two-layer neural network. *Neuron* 37 989–999. 12670427 10.1016/s0896-6273(03)00149-1

[B35] Polsky A. Mel B. W. Schiller J. (2004). Computational subunits in thin dendrites of pyramidal cells. *Nat. Neurosci.* 7:621. 10.1038/nn1253 15156147

[B36] Ponomarenko N. Jin L. Ieremeiev O. Lukin V. Egiazarian K. Astola J. (2015). Image database tid2013: Peculiarities, results and perspectives. *Signal Process.* 30 57–77.

[B37] Ponomarenko N. Lukin V. Zelensky A. Egiazarian K. Carli M. Battisti F. (2009). TID2008 - a database for evaluation of full-reference visual quality assessment metrics. *Adv. Modern Radioelectr.* 10 30–45. 10.1109/TIP.2015.2439035 26054063

[B38] Rimac-Drlje S. Vranjes M. Zagar D. (2009). “Influence of temporal pooling method on the objective video quality evaluation,” in *2009 IEEE International Symposium on Broadband Multimedia Systems and Broadcasting*, (Bilbao: IEEE), 1–5.

[B39] Rodrigues Y. E. Tigaret C. M. Marie H. O’Donnell C. Veltz R. (2021). A stochastic model of hippocampal synaptic plasticity with geometrical readout of enzyme dynamics. *Biorxiv* [Preprint]. 10.1101/2021.03.30.437703 PMC10435238 37589251

[B40] Seshadrinathan K. Bovik A. C. (2009). Motion tuned spatio-temporal quality assessment of natural videos. *IEEE Trans. Image Process.* 19 335–350. 10.1109/TIP.2009.2034992 19846374

[B41] Seshadrinathan K. Soundararajan R. Bovik A. C. Cormack L. K. (2010a). Study of subjective and objective quality assessment of video. *IEEE Trans. Image Process.* 19 1427–1441. 20129861 10.1109/TIP.2010.2042111

[B42] Seshadrinathan K. Soundararajan R. Bovik A. C. Cormack L. K. (2010b). A subjective study to evaluate video quality assessment algorithms. *Hum. Vis. Electr. Imaging* 7527 128–137.

[B43] Sheikh H. R. Sabir M. F. Bovik A. C. (2006). A statistical evaluation of recent full reference image quality assessment algorithms. *IEEE Trans. Image Process.* 15 3440–3451. 17076403 10.1109/tip.2006.881959

[B44] Sheikh H. Bovik A. (2006). Image information and visual quality. *IEEE Trans. Image Process.* 15 430–444. 10.1109/TIP.2005.859378 16479813

[B45] Silver R. A. (2010). Neuronal arithmetic. *Nat. Rev. Neurosci.* 11 474–489. 20531421 10.1038/nrn2864 PMC4750293

[B46] Soundararajan R. Bovik A. C. (2012). Video quality assessment by reduced reference spatio-temporal entropic differencing. *IEEE Trans. Circ. Syst. Video Technol.* 23 684–694. 10.1109/TIP.2011.2166082 21878414

[B47] Video Quality Experts Group (2010). *Report on the validation of video quality models for high definition video content.* Available online at: http://www.vqeg.org/ (accessed April 14, 2023).

[B48] Vilankar K. P. Field D. J. (2017). Selectivity, hyperselectivity, and the tuning of V1 neurons. *J. Vis.* 17:9. 10.1167/17.9.9 28813565

[B49] Wang Z. Bovik A. C. (2002). A universal image quality index. *IEEE Signal Process. Lett.* 9 81–84. 10.1109/97.995823

[B50] Wang Z. Simoncelli E. P. (2005). “Translation insensitive image similarity in complex wavelet domain,” in *Proceedings of IEEE International Conference on Acoustics, Speech, and Signal Processing* (IEEE), (Piscataway, NJ: IEEE). 10.1109/TIP.2008.926161

[B51] Wang Z. Bovik A. Sheikh H. Simoncelli E. (2004). Image quality assessment: From error visibility to structural similarity. *IEEE Trans. Image Process.* 13 600–612. 819861 10.1109/TIP.2003.819861 15376593

[B52] Wang Z. Simoncelli E. Bovik A. (2003). Multiscale structural similarity for image quality assessment. *Thrity Seventh Asilomar Conf. Signals Syst. Comput.* 2 1398–1402.

[B53] Xue W. Zhang L. Mou X. Bovik A. C. (2014). Gradient magnitude similarity deviation: A highly efficient perceptual image quality index. *IEEE Trans. Image Process.* 23 684–695. 10.1109/TIP.2013.2293423 26270911

[B54] Zhang F. Mackin A. Bull D. R. (2017). “A frame rate dependent video quality metric based on temporal wavelet decomposition and spatiotemporal pooling,” in *2017 IEEE International Conference on Image Processing (ICIP)*, (Piscataway, NJ: IEEE), 300–304. 10.1109/ICIP.2017.8296291

[B55] Zhang L. Shen Y. Li H. (2014). VSI: A visual saliency-induced index for perceptual image quality assessment. *IEEE Trans. Image Process.* 23 4270–4281. 10.1109/TIP.2014.2346028 25122572

[B56] Zhang L. Zhang L. Mou X. Zhang D. (2011). FSIM: A feature similarity index for image quality assessment. *IEEE Trans. Image Process.* 20 2378–2386. 10.1109/TIP.2011.2109730 21292594

[B57] Zhang R. Isola P. Efros A. A. Shechtman E. Wang O. (2018). “The unreasonable effectiveness of deep features as a perceptual metric,” in *Proceedings of the IEEE conference on computer vision and pattern recognition*, (Piscataway, NJ: IEEE), 586–595. 10.1109/CVPR.2018.00068

